# Comparison of Adjuvant Clindamycin vs Linezolid for Severe Invasive Group A *Streptococcus* Skin and Soft Tissue Infections

**DOI:** 10.1093/ofid/ofad588

**Published:** 2023-11-24

**Authors:** Emily L Heil, Harpreet Kaur, Anthony Atalla, Sapna Basappa, Minu Mathew, Hyunuk Seung, J Kristie Johnson, Gregory M Schrank

**Affiliations:** Department of Practice, Sciences, and Health Outcomes Research, University of Maryland School of Pharmacy, Baltimore, Maryland, USA; Department of Medicine, Division of Infectious Diseases, University of Maryland School of Medicine, Baltimore, Maryland, USA; University of Maryland School of Medicine, Baltimore, Maryland, USA; Department of Pharmacy, University of Massachusetts Memorial Medical Center, Worcester, Massachusetts, USA; Department of Medicine, Milton S. Hershey Medical Center, Hershey, Pennsylvania, USA; Department of Practice, Sciences, and Health Outcomes Research, University of Maryland School of Pharmacy, Baltimore, Maryland, USA; Department of Pathology, University of Maryland School of Medicine, Baltimore, Maryland, USA; Department of Medicine, Division of Infectious Diseases, University of Maryland School of Medicine, Baltimore, Maryland, USA

**Keywords:** clindamycin, group A *Streptococcus*, linezolid, necrotizing fasciitis, toxin

## Abstract

**Background:**

Linezolid may be an option for severe group A *Streptococcus* (GAS) infections based on its potent in vitro activity and antitoxin effects, but clinical data supporting its use over clindamycin are limited. This study evaluated treatment outcomes in patients with severe GAS skin and soft tissue infections who received either linezolid or clindamycin.

**Methods:**

This retrospective single-center cohort study examined patients with GAS isolated from blood and/or tissue cultures with invasive soft tissue infection or necrotizing fasciitis who underwent surgical debridement and received linezolid or clindamycin for at least 48 hours. The primary outcome was percentage change in Sequential Organ Failure Assessment (SOFA) score from baseline through 72 hours of hospitalization.

**Results:**

After adjustment for time to first surgical intervention among patients with a baseline SOFA score >0 (n = 23 per group), there was no difference in reduction of SOFA score over the first 72 hours in patients receiving clindamycin vs linezolid. In the entire cohort (n = 26, clindamycin; n = 29, linezolid), there was no difference in inpatient mortality (2% vs 1%) or any secondary outcomes, including duration of vasopressor therapy, intensive care unit length of stay, and antibiotic-associated adverse drug events.

**Conclusions:**

There was no difference in reduction of critical illness as measured by SOFA score between baseline and 72 hours among patients treated with clindamycin vs linezolid. Given its more favorable side effect profile, linezolid may be a viable option for the treatment of serious GAS infections and should be further studied.

## Teaser

There was no difference in the reduction of critical illness as measured by the Sequential Organ Failure Assessment score between baseline and 72 hours among patients treated with linezolid vs clindamycin for severe invasive group A streptococcal infections.

*Streptococcus pyogenes*, also known as group A *Streptococcus* (GAS), can cause serious infections, such as streptococcal toxic shock syndrome and necrotizing fasciitis, due in part to superantigens, including streptococcal pyrogenic exotoxins [1]. These superantigens can cause circulatory shock and multisystem organ failure, which significantly increase the risk of mortality in GAS necrotizing soft tissue infections [2–4]. Bacterial protein synthesis inhibitors can suppress the production of bacterial exotoxins and dampen the toxin-induced host inflammatory response [4]. Current guidelines by the Infectious Diseases Society of America recommend clindamycin plus penicillin for the treatment of GAS causing necrotizing fasciitis and toxic shock syndrome, but there is potential benefit beyond those syndromes [5, 6]. Clindamycin is associated with several adverse effects, such as *Clostridioides difficile* infection and gastrointestinal upset, and there are increasing rates of clindamycin resistance in gram-positive organisms, such as beta-hemolytic streptococci [7–9]. Linezolid is an alternative option for GAS infections based on its potent in vitro activity against streptococcal species and antitoxin effects and may be associated with fewer adverse effects when administered for short treatment courses [4, 10]. In vitro data have shown that clindamycin and linezolid, when used alone or in combination with penicillin, can reduce exotoxin production and thus optimize treatment of GAS infections [11]. However, clinical evidence for the use of linezolid in GAS infections is limited to case reports and a recent single-center retrospective cohort that was not specific to GAS [12, 13]. An increased incidence of invasive group A streptococcal infections has been reported in the past few years, adding urgency to the need to determine optimal antimicrobial therapy for these infections [14–16].

Our institution has a dedicated skin and soft tissue surgical service that sees a high volume of patients with severe skin and soft tissue infections, including referrals throughout the region. The purpose of this study was to evaluate treatment outcomes in a cohort of patients with severe invasive skin and soft tissue infections caused by GAS who received either linezolid or clindamycin as part of their antibiotic treatment regimen.

## METHODS

This was a retrospective single-center cohort study of patients admitted to the R. Adams Cowley Shock Trauma Center at the University of Maryland Medical Center with GAS isolated from blood and/or tissue cultures obtained after admission from January 2017 through March 2023. Adult patients aged ≥18 years with invasive soft tissue infection or necrotizing fasciitis were included in the study if they had GAS isolated from a normally sterile site (blood, other sterile fluid/tissue), underwent surgical debridement of their infection, and received either clindamycin or linezolid for at least 48 hours as part of their antibiotic treatment regimen [17, 18]. Patients were excluded if they received clindamycin and linezolid for >1 dose.

### Patient Consent Statement

The study was approved through the institutional review board of the University of Maryland, Baltimore, with a waiver of patient consent.

The primary outcome was the percentage change in Sequential Organ Failure Assessment (SOFA) score from baseline at hospital admission through 72 hours between patients who received clindamycin- and linezolid-containing antibiotic regimens to evaluate the anticipated physiologic impact of reducing circulating streptococcal exotoxin [19]. Secondary outcomes were inpatient mortality, duration of vasopressor requirement, intensive care unit (ICU) length of stay, ventilator days, hospital length of stay, adverse drug events attributed to the antibiotics of interest as documented in the medical record—specifically, hypersensitivity, *C difficile* infection based on positive polymerase chain reaction and confirmatory toxin test results plus receipt of targeted treatment, thrombocytopenia, and serotonin syndrome—and rates of clindamycin resistance.

Data were extracted from the electronic medical record and collected in REDCap (version 12.2.11). Data were collected on baseline demographics, SOFA scores at baseline time of admission and every 12 hours out to 72 hours [19], Laboratory Risk Indicator for Necrotizing Fasciitis (LRINEC) score at baseline [20], site of infection, total antibiotic duration, time to first surgical intervention, and number of surgical procedures. Baseline immunosuppression was defined as patients who were receiving chronic systemic steroids, solid organ transplant, stem cell transplant, active chemotherapy, or other lymphodepleting therapies or those who had HIV with a CD4 count ≤200 cells/mm^3^.

Susceptibilities are not routinely performed at our institution for GAS. To evaluate clindamycin and linezolid susceptibilities, clinical isolates were identified from patients in the study cohort that were available in our institution's biorepository. Frozen isolates were subbed to trypticase soy agar with 5% sheep blood agar plates and incubated overnight at 37 °C. Antimicrobial susceptibilities were performed with Senititre *Streptococcus* species STP6F Microbroth dilution plates (Thermo Fisher Scientific) following the manufacturer’s protocol. Minimal inhibitory concentration and interpretation were determined per the Clinical and Laboratory Standards Institute [21, 22].

Bivariate analysis of baseline characteristics and antibiotic treatment was performed with Fisher exact, chi-square, and Mann-Whitney *U* tests, as were the associations between outcomes and antibiotic treatment group. The frequency distribution in LRINEC scores across the clindamycin and linezolid groups was done with the chi-square test. A linear mixed model was used to analyze the percentage change in SOFA scores over 12-hour intervals between the antibiotics groups, adjusting for time to first surgical intervention as determined a priori. The random and fixed effects were estimated per the restricted maximum likelihood method. Patients with a baseline SOFA score of 0 were excluded from the model. Analyses were performed with SAS version 9.4 (SAS Institute).

## RESULTS

A total of 221 patients had GAS from blood and/or tissue cultures during the study, of which 55 were included in the analysis: 26 treated with clindamycin and 29 treated with linezolid as part of their antibiotic treatment regimen (Table 1). Most patients were excluded because they received clindamycin or linezolid for <48 hours, received both antibiotics, or received neither. Patient characteristics were generally well balanced between the groups; there was no difference in the frequency distribution of LRINEC scores (median, 7 in the clindamycin group vs 8 in the linezolid group; *P* = .7), although baseline SOFA score was higher in the clindamycin group but not statistically different (5 vs 2, *P* = .08). The majority of patients had LRINEC scores ≥6 in both groups, indicating the high likelihood of necrotizing fasciitis and risk of mortality (19/26 [73%] for clindamycin vs 21/29 [72%] for linezolid, *P* = .7) [20]. Across all patients, the median time from admission to first surgical intervention was 4.6 hours and did not differ between antibiotic groups (6 hours for clindamycin vs 4 hours for linezolid, *P* = .7).

**Table 1.** Patient Characteristics

	No. (%) or Median (IQR)			
Characteristic	Total (n = 55)	Clindamycin (n = 26)	Linezolid (n = 29)	*P* Value
Age, y^a^	50 (18.0)	49.3 (19.0)	50.6 (17.4)	.8
Male sex	38 (69.1)	16 (61.5)	22 (75.9)	.3
Transferred from an outside facility	31 (56.4)	17 (35.4)	14 (48.3)	.2
Body mass index				.2
<35	49 (89.1)	25 (96.2)	24 (82.8)	
≥35	6 (10.9)	1 (3.9)	5 (17.2)	
Baseline serum creatinine, mg/dL				.2
≤1.6	35 (63.6)	19 (73.1)	16 (55.2)	
>1.6	20 (36.4)	7 (26.9)	13 (44.8)	
Baseline score				
LRINEC	7 (4–9)	7 (4–9)	8 (5–9)	.7
SOFA	3 (1–8)	5 (2–8)	2 (1–5)	.08
Comorbidities				
Diabetes	13 (23.6)	4 (15.4)	9 (31.0)	.2
Chronic kidney disease	9 (16.4)	4 (15.4)	5 (17.2)	>.99
Peripheral vascular disease	5 (9.1)	1 (3.9)	4 (13.8)	.4
Substance use disorder	32 (58.2)	17 (65.4)	15 (51.7)	.3
History of tobacco use	26 (47.3)	10 (38.5)	16 (55.2)	.2
Immunocompromised	4 (7.3)	2 (7.7)	2 (6.9)	>.99
Trauma as inciting factor of infection	6 (10.9)	1 (3.9)	5 (17.2)	.2
Site of infection				
Head and neck	3 (5.5)	3 (11.5)	0	.1
Perineum or genitals	3 (5.5)	0	3 (10.3)	.2
Abdominal	0	0	0	>.99
Thorax trunk	4 (7.3)	3 (11.5)	1 (3.5)	.3
Extremity	46 (83.6)	20 (76.9)	26 (89.7)	.3
Inguinal	5 (9.1)	2 (7.7)	3 (10.3)	>.99
Organisms isolated from clinical cultures other than GAS				
Polymicrobial infection	27 (49.1)	12 (46.2)	15 (51.7)	.7
*Clostridium* spp	0	0	0	>.99
MSSA	12 (21.8)	5 (19.2)	7 (24.1)	.7
MRSA	11 (20)	4 (15.4)	7 (24.1)	.5
Gram negative bacilli	7 (12.7)	3 (11.5)	4 (13.8)	>.99
Anaerobes	1 (1.8)	0	1 (3.5)	>.99
Coagulase-negative *Staphylococcus*	8 (14.5)	2 (7.7)	6 (20.6)	.3
Adjunct therapies and management				
Time from admission to first surgery, h	4.6 (2.0–16.0)	6.0 (1.7–18.0)	4.0 (2.0–14.5)	.7
Hyperbaric oxygen	30 (54.6)	15 (57.7)	15 (51.7)	.7
IVIG given	12 (21.8)	8 (30.8)	4 (13.8)	.1
Duration of antitoxin therapy, d	3.3 (2.3–4.6)	2.7 (2.3,4.3)	3.5 (2.5–5.5)	.4
Vasopressors				
Norepinephrine	22 (40)	14 (53.9)	8 (27.6)	.06
Epinephrine	7 (12.7)	2 (7.7)	5 (17.2)	.4
Phenylephrine	2 (3.6)	1 (3.9)	1 (3.5)	>.99
Vasopressin	7 (12.7)	5 (19.2)	2 (6.9)	.2
None	32 (58.2)	12 (46.2)	20 (69.0)	.09

Abbreviations: GAS, group A *Streptococcus*; IVIG, intravenous immunoglobulin; LRINEC, Laboratory Risk Indicator for Necrotizing Fasciitis; MRSA, methicillin-resistant *Staphylococcus aureus*; MSSA, methicillin-sensitive *Staphylococcus aureus*; SOFA, Sequential Organ Failure Assessment.

^a^Mean (SD).

**Figure 1.**
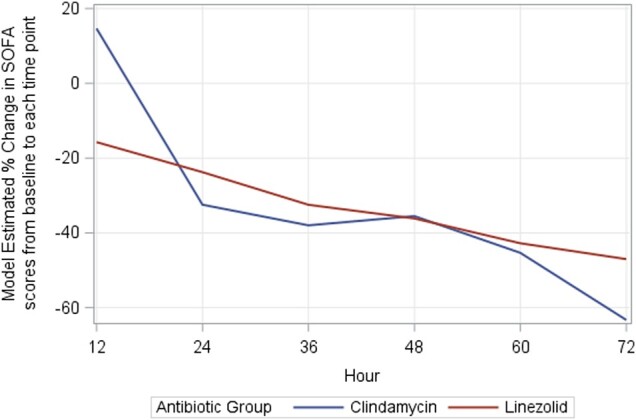
The model-estimated percentage change in Sequential Organ Failure Assessment (SOFA) score for clindamycin and linezolid across the first 72 hours of hospitalization (Table 3).

After adjustment for the time to first surgical intervention among patients with a baseline SOFA score >0 (n = 23 in both groups), there was no statistical difference in the reduction in SOFA score over the first 72 hours in patients receiving clindamycin vs linezolid (Table 2, Figure 1). At each measured 12-hour interval, the relative change in SOFA score was similar between treatment groups, with a consistent decline indicative of clinical improvement. The percentage change from baseline to 72-hour SOFA score was −61.4% in the clindamycin group as compared with −48.4% in the linezolid group (*P* = .4).

**Table 2.** Change in SOFA Scores From Baseline Across Various Time Points

	LS Mean (SE)		Clindamycin vs Linezolid		
SOFA Score	Clindamycin (n = 23)	Linezolid (n = 23)	LS Mean Difference (SE)	95% CI	*P* Value
Baseline	6.37 (0.85)	4.41 (0.85)	1.97 (1.09)	−0.47 to 4.41	.1
Percentage change to					
12 h	16.5 (22.8)	−17.1 (7.4)	33.6 (24.1)	−14.9 to 82.2	.2
24 h	−30.6 (9.6)	−25.1 (11.6)	−5.5 (15.2)	−36.3 to 25.3	.7
36 h	−36.1 (11.3)	−33.8 (12.4)	−2.3 (16.9)	−36.4 to 31.9	.9
48 h	−33.6 (14.5)	−37.5 (11.7)	3.9 (18.7)	−33.9 to 14.6	.8
60 h	−43.4 (13.6)	−44.1 (10.2)	0.7 (17.1)	−33.8 to 35.2	>.99
72 h	−61.4 (7.8)	−48.4 (11.6)	−13.0 (14.1)	−41.6 to 15.5	.4

Patients with a baseline SOFA score of 0 were excluded from this analysis.

Abbreviations: LS, least squares; SOFA, Sequential Organ Failure Assessment.

In the entire cohort, there was no difference in the rates of inpatient mortality between patients who received clindamycin and linezolid (2% vs 1%, respectively; *P* = .6). Two patients in the linezolid group were discharged to hospice; however, if they were in the deceased group, there would still be no statistically significant difference in mortality rates between agents (*P* = .6). Similarly, there were no statistically significant differences in other secondary outcomes, such as duration of vasopressor therapy, ventilator days, ICU length of stay, and hospital length of stay (Table 3). Adverse events were rare, with no cases of hypersensitivity reactions or serotonin syndrome identified and with low rates of *C difficile* infection, which did not differ between groups.

**Table 3.** Outcome Distribution of Patients Treated With Clindamycin vs Linezolid

	No. (%) or Median (IQR)			
Outcome	Total (n = 55)	Clindamycin (n = 26)	Linezolid (n = 29)	*P* Value
Inpatient mortality	3 (5.5)	2 (7.7)	1 (3.4)	.6
Amputation	10 (18.2)	4 (15.4)	6 (20.7)	.7
No.	23	14	9	
Time requiring vasopressors, h	41.4 (22.5–83.4)	42.1 (22.5–66.8)	39.1 (30.0–151.2)	.7
No.	26	17	9	
Ventilator days	2 (1–5)	2 (1–4)	4 (2–6)	.2
No.	27	16	11	
Length of stay, d				
Intensive care unit	8.8 (2.6–13.8)	8.2 (3.0–13.0)	9.5 (2.3–16.8)	.6
Hospital	12 (9–24)	14 (9–31)	10 (8–20)	.05
Adverse event				
*Clostridioides difficile* during hospital admission	2 (3.6)	1 (3.9)	1 (3.5)	>.99
Antibiotic-related thrombocytopenia	1 (1.8)	0	1 (3.5)	>.99

Seventeen isolates were identified in the biorepository and had antibiotic susceptibilities performed. All were susceptible to penicillin, levofloxacin, ceftriaxone, cefotaxime, daptomycin, chloramphenicol, meropenem, ertapenem, linezolid, cefepime, and vancomycin. Two isolates were resistant to tetracycline, erythromycin, and azithromycin, and one of these was also resistant to clindamycin. The clindamycin-resistant isolate was from a patient treated with clindamycin who had a successful treatment outcome and was discharged to a skilled nursing facility.

## DISCUSSION

In this comparison of patients treated with adjuvant clindamycin or linezolid for severe invasive group A streptococcal soft tissue infections, we found no difference between the antibiotics in the relative change in SOFA score over the first 72 hours following admission. Similarly, we did not identify any significant difference in any clinical secondary outcomes, such as duration of vasopressor therapy, ventilator days, or ICU length of stay. This study—the largest to date directly comparing clinical outcomes between these agents for invasive GAS—demonstrates similar clinical outcomes between clindamycin and linezolid and provides important findings that can inform treatment decisions for these highly morbid infections.

According to retrospective data, adjunctive use of clindamycin with a beta-lactam antibiotic for invasive GAS infections is associated with decreased in-hospital mortality as compared with beta-lactam antibiotics alone [6, 23]. However, primarily due to clindamycin's association with adverse events, including *C difficile* infection, alternative toxin-targeting adjunct agents for invasive GAS infections are needed. Use of linezolid as an alternative to clindamycin as part of an empiric regimen has the benefit of ensuring simultaneous effective antistreptococcal and staphylococcal therapy with adjuvant toxin-directed treatment. Thus, a more parsimonious regimen with fewer agents can be utilized by providers, sparing vancomycin or daptomycin that is often used in combination with clindamycin. Emerging literature suggests that linezolid-associated drug-drug interactions leading to serotonin syndrome are quite rare; furthermore, given the typical short durations of toxin-directed therapy that are utilized for GAS soft tissue infections, the risk of cytopenia and other linezolid-associated toxicities is low [24, 25]. Linezolid-containing regimens therefore offer the potential for an overall lower risk of adverse drug effects with similar effectiveness at mitigating the physiologic impact of streptococcal exotoxin.

In vitro models have shown that clindamycin and linezolid, when used alone or in combination with penicillin, can significantly reduce streptococcal pyrogenic exotoxin A production by *S pyogenes* [11]. Linezolid was highly efficacious in the treatment of severe murine myonecrosis caused by clindamycin-susceptible and clindamycin-resistant GAS [26]. However, clinical data are limited that support the use of linezolid as an adjunctive treatment in GAS infections. Dorazio et al published a study comparing results from a treatment protocol for necrotizing skin and soft tissue infections, before and after their institution substituted linezolid for clindamycin and vancomycin [13]. They found no difference in rates of 30-day mortality or *C difficile* infection between the groups, although a composite outcome of death, acute kidney injury, and *C difficile* infection was more common in the preintervention group with clindamycin vs the postintervention group with linezolid. Unfortunately, just 5 patients in their preintervention group and 3 in the postintervention group had cultures positive for GAS, limiting the conclusions as the data for toxin inhibitors for adjunct therapy are primarily rooted in GAS [13]. Our study was strengthened by including only culture-positive invasive GAS infections. Additional strengths were examining numerous clinical effectiveness outcomes, such as relative change in SOFA score and duration of vasopressor dependency, as a reflection of the proposed mechanistic benefit of these agents rather than a focus on adverse drug events. Our study thus provides valuable data to treating providers when considering the optimal antimicrobial regimen for patients with invasive GAS infections, as well as reassurance that linezolid demonstrates similar effectiveness as an adjuvant toxin-targeting agent as compared with clindamycin.

Rates of clindamycin resistance are increasingly reported in invasive GAS infections: >20% in surveillance data through 2017 and as high as 34.7% in surveillance data from the Centers for Disease Control and Prevention [8, 15]. The impact of clindamycin resistance on its clinical activity in an adjunct toxin-directed role is unclear, as murine models have shown that clindamycin still has activity even in the setting of clindamycin resistance [27]. Additionally, clindamycin-resistant GAS may exhibit decreased virulence factors, making an adjunctive antitoxin agent unnecessary. A murine model of GAS infections with penicillin treatment showed that 12-day survival was more likely in mice infected with clindamycin-resistant GAS than clindamycin-susceptible GAS [26]. Our limited evaluation of microbiologic data demonstrated overall low rates of resistance to any of the drugs tested, although only a minority of the patient isolates were available for testing and just 1 case of clindamycin resistance; as such, we are unable to draw any conclusions on the impact of clindamycin nonsusceptibility on its toxin inhibition. One case of thrombocytopenia was reported by the treating team in the medical chart as being partially attributed to linezolid.

Our study has several strengths—most notably, the inclusion only of patients with severe skin and soft tissue infections and confirmed *S pyogenes* in blood or operative cultures to establish a stronger causal inference between the adjuvant treatment selection and the outcome, as built on established in vitro research. We also believe that our selection of primary and secondary outcomes is a strength of our analysis and relevant for treating providers. Mortality is a frequently used outcome in studies evaluating therapeutic adjuncts for treatment such as intravenous immunoglobulin and antitoxin therapy but is infrequent in occurrence, thus restricting comparison due to a lack of statistical power; in addition, there is no standardized definition of clinical cure for serious GAS infections [13, 28, 29]. Considering the anticipated physiologic impact of reducing circulating streptococcal exotoxin, we chose the end point of evaluating change in SOFA score over time. Sequential measurement of SOFA has been used in critical care research to predict patient outcomes, and early changes are associated with mortality [30–32]. We therefore determined this to be an appropriate and measurable outcome of value for this retrospective comparison of antitoxin therapy. Another important consideration is that prompt surgical intervention is the cornerstone of management for necrotizing fasciitis, potentially more important than the antimicrobial selection itself; hence, controlling for time to first surgical intervention was determined a priori, and inclusion of these data strengthens the validity of our results.

This study has limitations, including the small sample size and retrospective design, which made it difficult to assess certain adverse effects of clindamycin, such as non–*C difficile* gastrointestinal intolerances; the inability to control for differences in surgical management; and the power to detect any differences in rare outcomes between the treatments, such as mortality. Although the groups were well balanced with respect to characteristics, comorbidity, and infection at the time of presentation, there is still potential for residual confounding. Notably, patients treated with clindamycin had higher rates of norepinephrine administration and higher baseline SOFA scores that were not statistically significant, which were accounted for by using the relative reduction in SOFA as the primary outcome with the exclusion of patients who had a SOFA score of 0. Additionally, given the role of our institution as a regional referral center for soft tissue infections, there may have been patients with invasive GAS infection admitted during the study period but not included in analysis due to the lack of microbiology data within our EHR, which was used to identify the cohort. Last, susceptibility testing for GAS is not routinely performed in our microbiology laboratory, so clindamycin resistance rates in our institution were unknown at the time when treatment selections were made, and our post hoc evaluation of susceptibilities was limited by which isolates were available in our biorepository.

Overall, in the largest study of its kind to date, there was no difference in the reduction of critical illness as measured by SOFA score between baseline and 72 hours when comparing patients treated with clindamycin vs linezolid with severe skin and soft tissue infections caused by GAS. Due to its generally more favorable side effect profile, linezolid may be a viable option for the treatment of serious GAS infections. However, this study should be considered exploratory, and prospective randomized studies would address some of the current limitations.

## Note

***Potential conflicts of interest.*** All authors: No reported conflicts.
